# Abnormal vital signs are strong predictors for intensive care unit admission and in-hospital mortality in adults triaged in the emergency department - *a prospective cohort study*

**DOI:** 10.1186/1757-7241-20-28

**Published:** 2012-04-10

**Authors:** Charlotte Barfod, Marlene Mauson Pankoke Lauritzen, Jakob Klim Danker, György Sölétormos, Jakob Lundager Forberg, Peter Anthony Berlac, Freddy Lippert, Lars Hyldborg Lundstrøm, Kristian Antonsen, Kai Henrik Wiborg Lange

**Affiliations:** 1 Department of Anaesthesia and Intensive Care, Hillerød Hospital, Hillerød, Denmark; 2 Department of Anaesthesia and Intensive Care, Aalborg Hospital, Aalborg, Denmark; 3 Department of Clinical Biochemistry, Hillerød Hospital, Hillerød, Denmark; 4 Deparment of Emergency Medicine, Hillerød Hospital, Hillerød, Denmark; 5 Emergency Medicine and Emergency Medical Services, Head Office, Capital Region of Denmark, Hillerød, Denmark

**Keywords:** Triage, Emergency Department, Database, Vital signs, Blood gas analysis

## Abstract

**Background:**

Assessment and treatment of the acutely ill patient have improved by introducing systematic assessment and accelerated protocols for specific patient groups. Triage systems are widely used, but few studies have investigated the ability of the triage systems in predicting outcome in the unselected acute population. The aim of this study was to quantify the association between the main component of the Hillerød Acute Process Triage (HAPT) system and the outcome measures; Admission to Intensive Care Unit (ICU) and in-hospital mortality, and to identify the vital signs, scored and categorized at admission, that are most strongly associated with the outcome measures.

**Methods:**

The HAPT system is a minor modification of the Swedish Adaptive Process Triage (ADAPT) and ranks patients into five level colour-coded triage categories. Each patient is assigned a triage category for the two main descriptors; vital signs, T_vitals_, and presenting complaint, T_complaint_. The more urgent of the two determines the final triage category, T_final_. We retrieved 6279 unique adult patients admitted through the Emergency Department (ED) from the Acute Admission Database. We performed regression analysis to evaluate the association between the covariates and the outcome measures.

**Results:**

The covariates, T_vitals_, T_complaint _and T_final _were all significantly associated with ICU admission and in-hospital mortality, the odds increasing with the urgency of the triage category. The vital signs best predicting in-hospital mortality were saturation of peripheral oxygen (SpO_2_), respiratory rate (RR), systolic blood pressure (BP) and Glasgow Coma Score (GCS). Not only the type, but also the number of abnormal vital signs, were predictive for adverse outcome. The presenting complaints associated with the highest in-hospital mortality were 'dyspnoea' (11.5%) and 'altered level of consciousness' (10.6%). More than half of the patients had a T_complaint _more urgent than T_vitals_, the opposite was true in just 6% of the patients.

**Conclusion:**

The HAPT system is valid in terms of predicting in-hospital mortality and ICU admission in the adult acute population. Abnormal vital signs are strongly associated with adverse outcome, while including the presenting complaint in the triage model may result in over-triage.

## Background

Emergency Departments (ED) are high-pressure health care settings, that experience a high number of visits. Overcrowding is an increasing global problem [1], and triage is a central process in prioritizing, when the resources are limited. This process is complex, and several triage scales have been designed to guide the clinician in prioritizing the patients, first in Australia, United Kingdom and Canada [2-4], and recently also in Sweden [5,6] and Denmark [7,8]. Most triage systems now operate with a five level colour-coded triage scale, where the patients are triaged after urgency from red (most urgent), through orange, yellow and green (not urgent) to blue (minor injuries). The assigned triage category is based on the vital signs and - in some of the systems - also on a presenting complaint algorithm [4,5,7]. Farrhoknia et al. have investigated the validity and reliability of the different triage scales in a systematic review [9]. They concluded that the scales used in the ED are supported by limited and often insufficient evidence. Furthermore there is a lack of studies investigating the ability of individual vital signs and presenting complaints to predict outcome in the group of acute ill patients admitted to the ED.

The aim of this study was

1) to quantify the association between the main components of the Hillerød Acute Triage (HAPT) triage system and the outcome measures; admission to Intensive Care Unit (ICU) and in-hospital mortality for patients admitted acutely through the ED.

2) to evaluate which vital signs, scored and categorized at admission that are most strongly associated with ICU admission and in-hospital mortality.

## Methods

**Figure 1:**
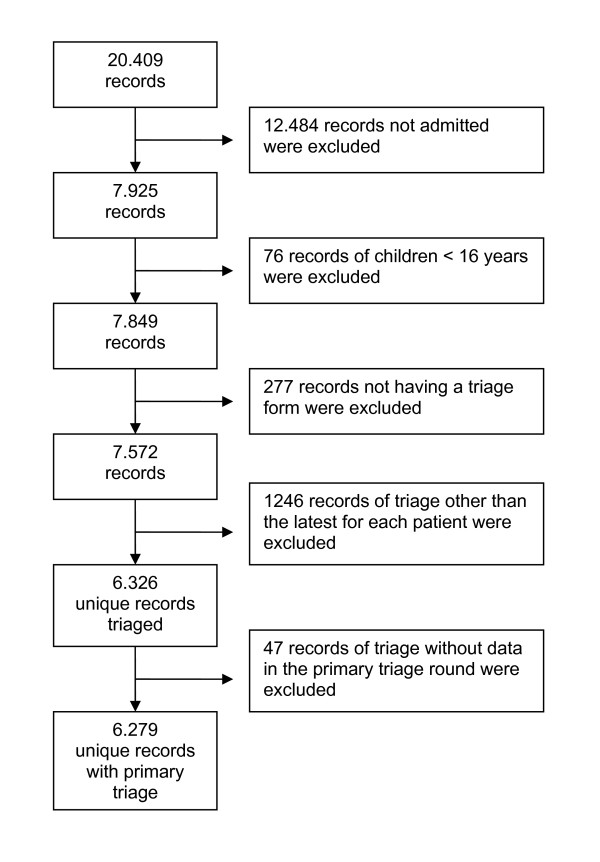
**Selection of the study cohort**. Patient records were excluded as explained in the figure. The final cohort included 6279 patients, representing the latest admission for every patient having a primary triage performed in the study period.

**Figure 2:**
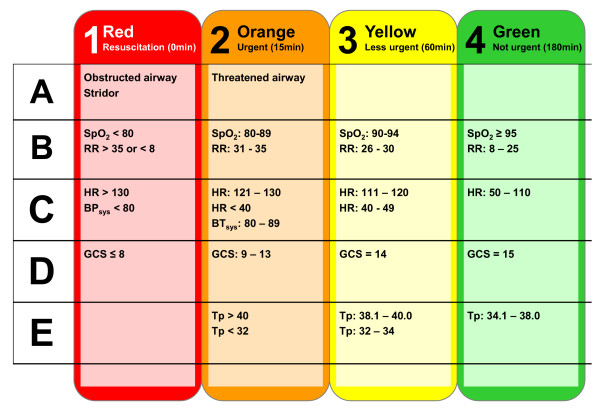
**Vital signs defining the colour-coded triage, T_vitals_**. RR: respiratory rate; SpO_2_: saturation of peripheral oxygen (pulse oxymetry); HR: heart rate; GCS: Glasgow Coma Score; Tp: temperature; ICU: Intensive Care Unit.

**Figure 3:**
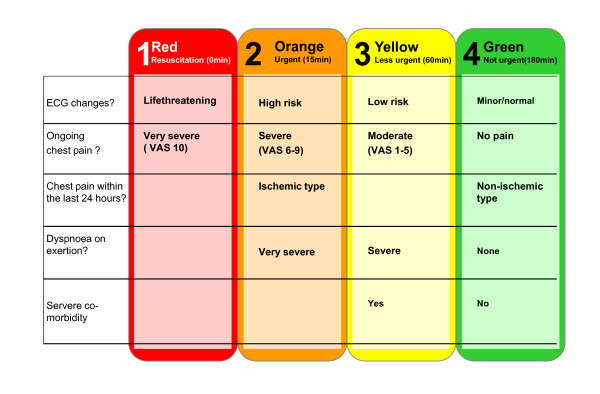
**Presenting complaint algorithm; chest pain**. ECG: Electrocardiography; VAS: Visual Analog Scale. Definitions of the terms used in the figure, e.g. 'ECG changes', 'chest pain of cardiac origin','functional dyspnoea' and 'risk patients' are found in the triage manual [7].

We retrieved 6279 unique patients from the Acute Admission Database in the period September 22, 2009 to February 28, 2010. Inclusion criteria were all patients aged more than 16 years admitted through the ED, either to the ED observationary unit or to a general ward. The cohort consisted of all patient referred from primary physicians, ambulance brought patients and self-referrals, who were admitted by the physician in charge after being triaged. Patients who fulfilled the criteria; 1) minor and isolated injury, 2) clinically unaffected, 3) no severe co-morbidity (e.g. diabetes), and 4) duration of symptoms less than 48 hours, were triaged as blue and excluded from the database, as they were not admitted to the ED or a general ward. Patients admitted on more than one occasion during the study period were only represented by the latest admission (Figure 1). Data included the initial assessment upon arrival, i.e. time and date, vital signs, presenting complaints and triage category. Furthermore we retrieved defined outcome measures; admission to ICU and in-hospital mortality. The formation and content of the Acute Admission Database is described in more detail by Barfod et al. [10]. Hillerød hospital is a 24-hour acute care hospital offering emergency, level-2 trauma, medical, surgical, and intensive care services for 310.000 citizens in North Zealand, Denmark. The ED has approximately 50.000 annual contacts. The triage system used at Hillerød Hospital is an adaptation of the Swedish Adaptive Process Triage (ADAPT) [5,6] and widely used in Denmark [8]. The system ranks patients into five colour-coded triage categories, consisting of red (immediate resuscitation, re-evaluation every 0 minutes (min)), orange (emergent, re-evaluation every 10 min), yellow (urgent, re-evaluation every 60 min), green (non-urgent, re-evaluation every 180 min) and blue (minor injuries or complaints, re-evaluation every 240 min). Each patient is assigned a triage category for the two main descriptors 1) Vital signs, T_vitals_, and 2) Presenting complaint, T_complaint_. T_vitals _is determined by scoring the vital signs according to Figure 2. The most abnormal vital signs define the T_vitals _category. T_complaint _is determined by choosing an algorithm matching the patients presenting complaint, e.g. chest pain, and then follow the algorithm to define the colour-coded triage category (Figure 3). The more urgent of T_vitals _or T_complaint _determines the final colour-coded triage category, T_final_, which in turn determines the level of patient observation and treatment.

The covariates retrieved for the current study were:

• Age.

• Gender.

• Time for contact; morning (08.00-15.59 hours), evening (16.00-23.59 hours), night (00.00-07.59 hours)

• Weekday (week-end versus week-day)

• Vital signs: SpO_2_, RR, systolic BP, heart rate (HR) and GCS. The vital signs were categorized according to the vital signs triage category, T_vitals _(Figure 2).

• Presenting complaint: Presenting complaint algorithms and the associated triage level, T_complaint_. An example of a presenting complaint algorithm for chest pain is shown in Figure 3.

• Final triage level, T_final_.

Outcome measures were

• Admission to ICU

• In-hospital mortality, i.e. mortality within the duration of the actual admission

The study was approved by The Danish National Committee on Biomedical Research Ethics, J.nr. H-A-2009-006, and the Danish Data Protection Agency, Copenhagen, J.nr. HIH 2009-2 Akutdatabasen.

### Statistical analysis

We analyzed the association between the main components of the triage system and the two endpoints; ICU admission and in-hospital mortality in a univariate regression analysis including the covariates: T_vitals_, T_complaint _and T_final_. Furthermore, we analyzed vital signs, categorized according to the triage model, as well as the effect of age, gender, time and weekday in a univariate regression analysis. Multivariate logistic regression analysis was then performed, including all significant covariates from the univariate regression analyses. We performed backward stepwise regression to identify the final model. Analyses of the interaction of the first order were performed between the covariates included in the multivariate model, and model control was performed by using the Hosmer and Lemeshow goodness-of-fit test. For evaluation of the agreement between the categorical classification of T_vitals _and T_complaint_, the weighted kappa value was estimated. The prevalence and pattern for missing values among the covariates were described. P < 0.05 was regarded as statistically significant. This study has been presented according to the Strengthening the Reporting of Observational Studies in Epidemiology statement [11].

## Results

Baseline characteristics, T_final _and vital signs for all patients are displayed in Table 1. Temperature measurements were excluded, as they were not an established part of the triage system at the time of data retrieval. The majority of patients (75%) had vital signs within the defined normal range, while 25% had one or more abnormal vital signs. The most common abnormal vital signs in the cohort were SpO_2 _(9.2%), GCS (6.6%) and RR (4.8%). The most common presenting complaint algorithms and those associated with the highest in-hospital mortality rates are shown in Table 2. Of the 15 presenting complaints associated with the highest mortality rates, 5 followed the algorithm 'abdominal complaint' and 4 followed the algorithm 'neurological complaint'. In 5.7% of the patients, the primary triage resulted in the score of 'no adequate presenting complaint algorithm'. Many patients (21%) only had a main presenting complaint assigned, e.g. abdominal complaint without specification, and could therefore not receive a colour-coding for the presenting complaint. In 6.0% of the patients, a presenting complaint assessment was missing.

**Table 1:** Patient characteristics

	ICU admission		In-hospital mortality			Missing
	ICU	no ICU	Dead	Alive	Total	of total
All patients	102	6177	107	6172	6279	
*Categorical Covariates*						
Gender						
Male	57 (55.9)	2974 (48.1)	56 (52.3)	2975 (48.2)	3031 (48.3)	0 (0)
Female	45 (44.1)	3203 (51.9)	51 (47.7)	3197 (51.8)	3248 (51.7)	
Tfinal						
Red	30 (29.4)	248 (4.0)	31 (29.0)	247 (4.0)	278 (4.4)	0 (0)
Orange	40 (39.2)	1544 (25.0)	36 (33.6)	1548 (25.1)	1584 (25.2)	
Yellow	26 (25.5)	2401 (38.9)	30 (28.0)	2397 (38.8)	2427 (38.7)	
Green	6 (5.9)	1984 (32.1)	10 (9.3)	1980 (32.1)	1990 (31.7)	
SpO_2 _(%)						
95-100	69 (73.4)	5440 (90.8)	64 (65.3)	5445 (90.9)	5509 (87.7)	191 (3.0)
90-94	13 (13.8)	429 (7.2)	19 (19.4)	423 (7.1)	442 (7.0)	
80-89	11 (11.7)	108 (1.8)	12 (12.2)	107 (1.8)	119 (1.9)	
< 80	1 (1.1)	17 (0.3)	3 (3.1)	15 (0.3)	18 (0.3)	
RR (min^-1^)						
> 35	3 (3.4)	35 (0.6)	4 (4.4)	34 (0.6)	38 (0.6)	785 (12.5)
31-35	4 (4.6)	49 (0.9)	6 (6.6)	47 (0.9)	53 (0.8)	
26-30	11 (12.6)	204 (3.8)	12 (13.2)	203 (3.8)	215 (3.4)	
8-25	68 (78.2)	5119 (94.7)	69 (75.8)	5118 (94.7)	5187 (82.6)	
< 8	1 (1.1)			1 (0.0)	1 (0.0)	
BP (mmHg)						
90-	93 (97.9)	6008 (99.1)	93 (90.3)	6008 (99.2)	6101 (97.2)	120 (1.9)
80-89	2 (2.1)	36 (0.6)	6 (5.8)	32 (0.5)	38 (0.6)	
< 80	0 (0.0)	20 (0.3)	4 (3.9)	16 (0.3)	20 (0.3)	
HR (min^-1^)						
> 130	9 (9.6)	111 (1.8)	4 (3.9)	116 (1.9)	120 (1.9)	126 (2.0)
121-130	12 (12.8)	111 (1.8)	6 (5.8)	117 (1.9)	123 (2.0)	
111-120	11 (11.7)	261 (4.3)	7 (6.8)	265 (4.4)	272 (4.3)	
50-110	60 (63.8)	5510 (90.9)	83 (80.6)	5487 (90.7)	5570 (88.7)	
40-49	1 (1.1)	56 (0.9)	2 (1.9)	55 (0.9)	57 (0.9)	
< 40	1 (1.1)	10 (0.2)	1 (1.0)	10 (0.2)	11 (0.2)	
GCS						
15	65 (73.0)	5472 (93.3)	63 (67.0)	5474 (93.4)	5537 (88.2)	327 (5.2)
14	9 (10.1)	209 (3.6)	10 (10.6)	208 (3.6)	218 (3.5)	
9-13	5 (5.6)	136 (2.3)	7 (7.4)	134 (2.3)	141 (2.2)	
≤ 8	10 (11.2)	46 (0.8)	14 (14.9)	42 (0.7)	56 (0.9)	
Admission day						
weekend	24 (25.3)	1416 (23.9)	21 (20.6)	1419 (23.9)	1440 (22.9)	247 (3.9)
weekday	71 (74.7)	4521 (76.1)	81 (79.4)	4511 (76.1)	4592 (73.1)	
Admission time						
day	41 (43.2)	3163 (53.3)	61 (59.8)	3143 (53.0)	3204 (51.0)	247 (3.9)
evening	33 (34.7)	1932 (32.5)	30 (29.4)	1935 (32.6)	1965 (31.3)	
night	21 (22.1)	842 (14.2)	11 (10.8)	852 (14.4)	863 (13.7)	
*Continuous covariates*						
Age	67 (17-93)	62 (16-108)	77 (29-99)	62 (16-108)		

The table shows number of patients. The figures in parentheses are the column percentages within each categorical covariate. For continuous covariates median and range are presented

ICU: intensive care unit; SpO_2 _: saturation of peripheral oxygen; RR: respiratory rate; BP: systolic blood pressure; HR: heart rate; GCS: Glasgow Coma Score; Admission time: day = 08.00-1559 hours; evening = 16.00-23.59 hours; night = 00.00-07.59 hours

**Table 2:** Presenting complaints

**Table 2.a**.	Most common presenting complaint			
		**N**	**N (%)**	**Mortality**

	Abdominal complaint	1265	**20.1**	3.1
	*Abdominal pain*	*768*	***12.2***	*2.5*
	Chest pain	611	**9.8**	1.5
	Dyspnoea	565	**9.0**	7.3
	Neurological	507	**8.1**	2.4
	Fever/unspecified infection	427	**6.8**	1.4
	No adequate category	360	**5.7**	5.0
	Extremity swelling/pain	339	**5.4**	0.3
	Cardiac complaints*	246	**3.9**	2.8
	Syncope	219	**3.5**	0.5
	Extremity injury	206	**3.3**	0.0
	Abnormal lab values	154	**2.5**	3.2
	Hip fracture	151	**2.4**	0.1
	Neck/back pain	147	**2.3**	0.7
	Headache	141	**2.3**	0.0

Table 2.b.	**Presenting complaints rated after mortality**			
		**N**	**N (%)**	**Mortality**

	*Dyspnoea, ns*	*113*	*1.8*	***11.5***
	Altered level of consciousness	94	1.5	**10.6**
				
	*Aphasia*	*64*	*1.0*	***7.8***
	*Vomiting blood*	*74*	*1.2*	***6.8***
	No relevant category	360	5.7	**5.0**
	*Cough*	*50*	*0.8*	***4.0***
	*Abdominal complaints, ns*	*108*	*1.7*	***3.7***
	*Blood in stool/melaena*	*137*	*1.8*	***3.6***
	Hypertension	55	*1.9*	**3.6**
	Abnormal lab values	154	*1.10*	**3.2**
	*Diarrhoea*	*64*	*1.11*	***3.1***
	*Neurological symptoms, ns*	*101*	*1.12*	***3.0***
	*Anaemia*	*70*	*1.13*	***2.9***
	*Unilateral extremity weakness*	*102*	*1.14*	***2.9***
				
	*Vomiting*	*72*	*1.15*	***2.8***

The 15 most common presenting complaints and the presenting complaints associated with the highest in-hospital mortality. Categories in *italics *are subcategories of a main category, while categories in plain text are one of the 29 algorithms for presenting compliant. ns: non specified. * neither categorized as chest pain nor syncope.

The main components of the triage system; T_vitals_, T_complaint _and T_final _were all significantly associated with ICU admission and in-hospital mortality in a univariate regression analysis.

For all three covariates, the odds increased substantially with the urgency of the triage category (Table 3). A multivariate analysis including the significant covariates from the univariate analysis, demonstrated T_vitals _as being the strongest predictor for in-hospital mortality, while T_complaint _was excluded due to lack of significance (Table 4). Table 5 illustrates the agreement between T_vitals _and T_complaint _in determining T_final_. The distribution of the patients is asymmetric; more patients are located above than below the diagonal of the table. This means, that patients are assigned a higher (more severe) triage category due to the presenting complaint than the corresponding vital signs. In 52.3% of the patients, T_final _was determined by the presenting complaint, and in just 6%, T_final _was determined by the vital signs. Agreement in the colour-coding was seen in 41.5%. A weighted kappa value of 0.20 (0.18-0.22) supports a poor agreement between the two covariates.

**Table 3:** Univariate analysis of the association between triage category and admission to ICU and in-hospital mortality

	Total	ICU	In-hospital mortality
	n	OR (95%CI)	OR (95% CI)
**Tvitals**			
Red	169	38.6** (20.9-71.4)	20.1** (11.4-35.5)
Orange	568	10.9** (6.0-19.7)	3.9** (2.3-6.8)
Yellow	1284	4.3** (2.4-7.8)	2.2# (1.3-3.8)
Green	4140	1.0	1.0
Missing	118 (1.9%)		
**Tcomplaint**			
Red	133	27.3** (8.8-84.9)	13.2** (5.0-34.6)
Orange	1290	5.0* (1.8-14.5)	4.6** (2.0-10.2)
Yellow	2043	3.1# (1.1-8.8)	2.2 (1.0-5.0)
Green	1028	1.0	1.0
No category	1408	7.1** (2.5-20.0)	6.0** (2.7-13.3)
Missing	377 (6.0%)		
**Tfinal**			
Red	272	40.3** (16.6-97.7)	24.0** (14.8-38.8)
Orange	1590	8.5** (3.6-20.2)	8.0** (5.2-12.3)
Yellow	2415	3.5** (1.5-8.7)	2.8** (1.8-4.4)
Green	2002	1.0	1.0
Missing	0 (0.0%)		

ICU: Intensive Care Unit; SpO_2 _: saturation of peripheral oxygen; RR: respiratory rate; BP: systolic blood pressure; HR: heart rate; GCS: Glasgow Coma Score; OR: odds ratio; CI: confidence interval; T_vitals _: Triage category determined by vital signs; T_complaint _: Triage category determined by presenting complaint algorithm. T_final _: final triage category.

** P < 0.0001, * P < 0.001, # P < 0.05

**Table 4:** The multivariate association between triage categories and in-hospital mortality

		OR (95% CI)	P
**Tvitals**	Red	6.57 (2.25-19.17)	< 0.001
	Orange	6.69 (3.29-13.58)	< 0.0001
	Yellow	2.98 (1.31-5.45)	< 0.001
	Green	1.0	
**Tfinal**	Red	5.43 (1.68-17.52)	< 0.005
	Orange	1.49 (0.62-3.56)	ns
	Yellow	1.34 (0.86-3.08)	ns
	Green	1.0	

T_vitals _: Triage category determined by vital signs

T_complaint_: Triage category determined by presenting complaint algorithm

OR: odds ratio; CI: confidence interval

**Table 5:** Agreement between T_vitals _and T_complaint_

			Tvitals		
	
Tcomplaint	Red	Orange	Yellow	Green	Total
**Red**	38	9	7	57	111
**Orange**	31	270	206	780	1287
**Yellow**	20	99	645	1283	2047
**Green**	7	18	98	907	1030

**Total**	96	396	956	3027	4475

T_vitals_: Triage category determined by vital signs

T_complaint_: Triage category determined by presenting complaint algorithm

The table illustrates complete cases, i.e. where both T_vitals _and T_complaint _were colour coded. A total of 1342 (21%) patients did have a presenting complaint assigned, but no colour coding due to insufficient information. T_complaint _was missing in 370 (5.9%) patients, while T_vitals _were missing in 118 (1.9%) patients.

To further elucidate the impact of T_vitals _on outcome, we analyzed the association between the categories of individual vital signs according to the triage model and the defined outcome measures. All significant covariates identified in the univariate analysis were included in a subsequent multivariate analysis, identifying age and the categorical variables; SpO_2_, RR, systolic BP and GCS as independent risk factors for in-hospital mortality (Table 6). With respect to ICU admission, age, SpO_2_, HR and GCS were independent risk factors. There were no significant interactions of first degree between the multivariate significant covariates concerning in-hospital mortality and admission to ICU. Our model testing indicates a robust multivariate model. Not only the type, but also the number of abnormal vital signs were strong predictors for in-hospital mortality and admission to ICU. Odds Ratios (OR) increased depending on the number of abnormal vital signs in the primary triage round (Table 7).

**Table 6:** Univariate association and multivariate model for prediction of ICU admission and in-hospital mortality

	Univariate association						Multivariate model					
	**Admission to ICU**			**In-hospital mortality**			**Admission to ICU**			**In-hospital mortality**		

	**OR**	**CI**	**P**	**OR**	**CI**	**P**	**OR**	**CI**	**P**	**OR**	**CI**	**P**

**SpO_2 _**(%)												
95-100	Reference			1.0			Reference			Reference		
90-94	2.35	1.31-4.20	< 0.01	4.78	3.17-7.22	< 0.0001	1.49	0.79-2.82	0.21	2.79	1.73-4.52	< 0.0001
80-89	7.49	3.86-14.51	< 0.0001	13.00	7.77-21.79	< 0.0001	3.76	1.78-7.96	0.001	3.73	1.89-7.37	< 0.0001
< 80	8.42	1.88-36.17	< 0.01	20.44	7.27-57.47	< 0.0001	5.48	1.16-25.84	0.03	9.01	2.18-37.26	0.002
**RR **(min^-1^)												
8-25	Reference			1.0						Reference		
26-30	3.76	1.97-7,20	< 0.0001	4.26	2.50-7.26	< 0.0001				1.89	0.10-3.58	0.052
31-35	5.92	2.08-16.87	< 0.001	10.55	5.01-22.21	< 0.0001				4.96	2.00-12.25	0.001
> 35	9.11	3.49-23.80	< 0.0001	14.82	7.13-30.82	< 0.0001				6.41	2.59-15.89	< 0.0001
**BP **(mmHg)												
90-	Reference			1.0						Reference		
80-89	4.97	1.50-16.38	< 0.01	12.26	5.73-26.4	< 0.0001				5.07	1.69-15.16	0.004
< 80	3.22	0.43-24.32	0.26	10.56	3.49-31.99	< 0.0001				3.87	0.73-20.71	0.113
**HR **(min^-1^)												
< 40	6.51	0.84-50.50	0.07	3.46	0.45-26.65	0.23						
40-49	1.41	0.20-10.33	0.74	2.32	0.72-7.53	0.16	2.2	0.51-9.41	0.29			
50-110	Reference			1.0			Reference					
111-120	3.83	2.05-7.18	< 0.0001	2.03	1.11-3.73	0.02	3.19	1.62-6.29	0.001			
121-130	8.91	4.68-16.95	< 0.0001	3.88	1.98-7.58	< 0.0001	6.17	3.51-10.83	< 0.0001			
> 130	8.14	4.07-16.28	< 0.0001	2.50	1.08-5.80	0.033						
**GCS**												
15	Reference			1.0			Reference			Reference		
14	3.57	1.82-7.00	< 0.0001	3.33	1.80-6.16	< 0.0001	2.77	1.32-5.75	0.006	1.56	0.73-3.39	0.253
9-13	2.02	0.73-5.61	0.18	6.32	3.57-11.18	< 0.0001	1.29	0.45-3.71	0.63	3.72	1.97-7.03	< 0.0001
< 8	5.21	5.24-24.91	< 0.0001	24.60	13.65-44.36	< 0.0001	4.93	1.29-12.64	0.001	10.97	4.90-24.56	< 0.0001
**Age **(yrs)	1.02	1.01-1.03	< 0.002	1.06	1.04-1.07	< 0.0001	1.01	1.00-1.02	0.048	1.05	1.03-1.06	< 0.0001

ICU: Intensive Care Unit, SpO_2 _: saturation of peripheral oxygen, RR: respiratory rate, BP: systolic blood pressure, HR: heart rate, GCS: Glasgow Coma Score

Reference: SpO_2 _= 95-100; RR = 8-25; BP > 90; HR 50-110; GCS = 15

Age is expressed as continuous covariate. The OR at e.g. 1.02 represents the increased risk of ICU admission being 1 year older

**Table 7:** The univariate association between the number of abnormal vital signs, ICU admission and in-hospital mortality

Abnormal vital signs (N)	Patients (N)	Patients (%)	Admission to ICU			In-hospital mortality		
			OR	CI	P	OR	CI	P
0	3916	74.9	1.0			1.0		

1	1002	19.2	2.2	1.25-3.89	0.006	4.03	2.57-6.32	< 0.0001

2	244	4.7	13.03	7.64-22.23	< 0.0001	12.37	7.43-20.58	< 0.0001

3	57	1.1	15.99	6.76-37.77	< 0.0001	29.36	14.66-58.81	< 0.0001

4	11	0.2	No ITA			37.27	9.53-145.77	< 0.0001

5	0	0						

ICU: intensive care unit; OR: odds ratio; CI: confidence interval

Illustrates cases with all 5 vital signs scored (SpO_2_, RR, BP, HR and GCS). A total of 1096 (20%) patients had one or more of the 5 vital signs missing in the primary triage

## Discussion

We have demonstrated the triage system and categories used as being valid in terms of predicting in-hospital mortality and ICU admission. The vital sign categories were strongly associated with adverse outcome, especially impaired GCS, RR and SpO_2_. The number of abnormal signs as well as the level of abnormality were important.

By including the presenting complaint triage category, the T_final _was up-graded in more than half of the patients. Hereby, the association between mortality and T_final _declined in comparison with a triage model only based on vital signs. However, in a clinical context it may be preferable to choose a high sensitivity at the expense of a low specificity. By including the presenting complaints in the model, priority is given to a patient with a potentially serious, although rare condition, who might present with normal vital signs. As an example, a very low proportion of patients with sudden, severe headache are diagnosed with a subarachnoid haemorrhage. These patients most often present with T_vitals _in green category (normal vital signs) but the presenting complaint results in orange category for T_complaint _and subsequently for T_final_.

In the triage model used, the greater the discrepancy from normal vital signs, the more urgent the triage category. For instance a patient with GCS 14 is assigned yellow triage category while a patient with GCS 7 is assigned red triage category. However, our results show that not only the grade of deviation from normality matters, but also the number and type of deviating vital signs should be taken into account, when risk assessing the patients. This finding favours triage systems using a score depending on the number of deviations like the Early Warning Score [12], Rapid Acute Physiology Score, [13] and the modified version for ED, Rapid Emergency Medicine Score REMS [14]. We found abnormal RR, SpO_2 _and GCS to be significant risk factors associated with adverse outcome. This is in accordance with a study by Olsson et al. [14], finding RR, coma and SpO_2 _to be significant covariates in a multivariate model for in-hospital mortality.

In our analysis, age was independently and significantly associated with outcome. Therefore age may be considered included in our future triage system, which already is the case for some other triage systems (e.g. Medical Emergency Triage and Treatment System, METTS [15]). We also found dyspnoea to be a common presenting complaint associated with high mortality. This is supported by our analysis, demonstrating abnormal RR and SpO_2 _to be strongly associated with adverse outcome.

There is no international consensus about which outcome variables should be used when evaluating different triage systems [16]. In a recent and extensive review of the literature about the evidence for using different triage scales, admission to hospital and mortality were used as proxy variables [9]. As all our patients were admitted to hospital, we chose ICU admission and in-hospital mortality as our outcome measures. Other endpoint may be of interest e.g. emergent operation, stroke or acute myocardial infarction, but none of these were evaluated in this study.

Neurological complaints were common and especially impaired consciousness and focal neurological signs were associated with high in-hospital mortality. More surprisingly perhaps, also gastro-intestinal complaints, especially vomiting of blood, melaena and diarrhoea were associated with high in-hospital mortality. Abdominal complaints (as a main group) accounted for 20.1% of the contacts and the group had an in-hospital mortality rate of 3.1%. In contrast, 'chest pain', although a common presenting complaint (9.8%), had a much lower in-hospital mortality rate in the present cohort (1.5%).

A major strength of the study is the prospective design and the inclusion of a large sample of consecutively retrieved, non-selected cohort of adult, acutely ill patients admitted to ED. All data were retrieved from the Acute Admission Database, minimizing risk of errors in data retrieval from separate databases.

There are several limitations in the present study. First of all the data do not include paediatric patients and highly specialized patients (i.e. neurosurgical, cardiovascular and major trauma) as these latter patients preferentially are admitted to highly specialized departments in other hospitals. Therefore the patients categorized in red triage category in our study are possibly not as ill as the highly specialized patients, that are transferred, and the difference between triage groups could therefore theoretically be even more pronounced, than we were able to show.

A further limitation is the missing data. Temperature measurement was not implemented by the time of retrieval of the cohort, and therefore not included in this study. Measurements of RR and GCS were missing in 12.5% and 5.2% in the primary triage round. The recording of these variables is subject to personal judgement as opposed to the automated measurement of BP and HR. In 5.6% of the patients no adequate triage category was scored for the presenting complaint, meaning that the patients were triaged, but it was not possible to find an adequate category for the presenting complaint. Other triage systems have a more comprehensive list of presenting complaints, for instance the Canadian Emergency Department Triage and Acuity Scale [17]. The span of severity within each group suggests that it is not the category itself that is suitable for risk assessment of the patient, but the triage colour emerging from the presenting complaint. A proportion of 21% of the patients (1342) were not given a colour-coding for the presenting complaint category due to insufficient information on the triage form. This is problematic because the patients with no colour-code in T_complaint _also had a significant risk of ICU admission or in-hospital mortality (Table 3). This should lead to a revision of the presenting complaints system to insure that all triaged patients are colour- coded.

In our study no evaluation of inter-observer agreement was done. This variation could however be significant [18]. Very few studies have assessed the inter-rater variability and the quality of the studies is poor [9].

The use of a triage system has the inherent risk of confounding by indication, since assignment of a triage category defines a level of observation and treatment. A patient assigned to the orange triage category is monitored and observed more closely than a patient assigned to the green triage category. Therefore the variation in mortality between the most and the least ill could theoretically be even more pronounced than we were able to show in our study.

The clinical implications of our findings are that most emphasis should be put on abnormal vital signs in the triage of the acutely ill patient, especially abnormal RR, SpO_2 _and impaired consciousness. Furthermore the groups of patients presenting with abdominal complaints, dyspnoea or neurological complaints should be analyzed further in order to identify patient profiles with high risk of adverse outcome.

## Conclusion

In conclusion, the triage system used (HAPT) is valid in terms of predicting ICU admission and in-hospital mortality in the acutely ill patients admitted through the ED. The most powerful predictors are abnormal vital signs, especially abnormal RR, SpO_2 _and GCS. Including the presenting complaint in the triage model generally results in over-triage of the patients when predicting ICU admission or in-hospital mortality.

## Abbreviations

ADAPT: Adaptive Process Triage; BE: base excess; BP: blood pressure; ED: Emergency Department; GCS: Glasgow Coma Score Scale; HAPT: Hillerød Adaptive Process Triage; ICU: Intensive Care Unit; LOS: length of stay; RR: respiratory rate; SpO_2_: saturation of peripheral oxygen (pulse oxymetry); T_complaint_: triage category determined by presenting complaint algorithm; T_final_: final triage category; T_vitals_: triage category determined by vital signs; Tp: temperature.

## Competing interests

The authors declare that they have no competing interests.

## Authors' contributions

CB participated in the conception and design of the database, the present study, data collection and interpretation, drafted and critically revised the manuscript. KL, ML and JD participated in the conception and design of the database, the present study, data collection and interpretation and critically revised the manuscript. PB, GS and KA participated en the conception and design of the database, data collection and interpretation and critically revised the manuscript. LL, FL and JLF participated in data interpretation and critically revised the manuscript. All authors read and approved the final version of the manuscript.

## References

[B1] Eitel DR Rudkin SE Malvehy MA Killeen JP Pines JM Improving service quality by understanding emergency department flow: a White Paper and position statement prepared for the American Academy of Emergency Medicine J Emerg Med 2010 38 1 70 79 10.1016/j.jemermed.2008.03.038 18514465

[B2] Meek R Phiri W Australasian Triage Scale: Consumer perspective Emerg Med Australas 2005 17 3 212 217 10.1111/j.1742-6723.2005.00725.x 15953221

[B3] Manchester Triage group Emergency triage: Manchester Triage Group 1997 London: BMJ Publishing Group, London, UK

[B4] Beveridge R Clarke B Janes N Canadian emergency department triage and acuity scale; implementation guidelines Can J Emerg Med 1999 1 2 28

[B5] Lethvall S ADAPT - Adaptiv Processtraige/VITALHISTORIER Version 1.1.2008. Giltiga 080424-090531 (Sweden)

[B6] Nordberg M Lethvall S Castrén M The validity of the triage system ADAPT Scand J Trauma Resusc Emerg Med 2010 18 Suppl 1 36 10.1186/1757-7241-18-S1-P3620565913

[B7] Lauritzen M Dahlin J Skriver C HAPT - Hilleroed Acute Process Triage 2011 http://www.hillerodhospital.dk/menu/Afdelinger/Akutafdelingen/Triage/Accessed august 24, 2011

[B8] Skriver C Lauritzen MP Forberg JL Gaardboe-Poulsen OB Mogensen CB Hansen CL Systematic process triage quickens the treatment of the most sick patients Ugeskr Laeger 2011 173 40 2490 2493 21975184

[B9] Farrohknia N Castren M Ehrenberg A Lind L Oredsson S Jonsson H Emergency department triage scales and their components: a systematic review of the scientific evidence Scand J Trauma Resusc Emerg Med 2011 30 19 42 21718476 10.1186/1757-7241-19-42 PMC3150303

[B10] Barfod C Lauritzen MMP Danker JK Sölétörmos G Forberg JL Berlac PA The formation and design of 'The Acute Admission Database' - a database including a prospective, observational cohort of 6279 patients triaged in the Emergency Department in a larger Danish hospital Scand J Trauma Resusc Emerg Med 2012 in press 10.1186/1757-7241-20-29 PMC3403899 22490233

[B11] von Elm E Altman DG Egger M Pocock SJ Gotzsche PC Vandenbroucke JP The Strengthening the Reporting of Observational Studies in Epidemiology (STROBE) statement: guidelines for reporting observational studies Rev Esp Salud Publica 2008 82 3 251 259 18711640 10.1590/s1135-57272008000300002

[B12] Prytherch DR Smith GB Schmidt PE Featherstone PI ViEWS-Towards a national early warning score for detecting adult inpatient deterioration Resuscitation 2010 81 8 932 937 10.1016/j.resuscitation.2010.04.014 20637974

[B13] Rhee KJ Fisher CJ Jr Willitis NH The Rapid Acute Physiology Score Am J Emerg Med 1987 5 4 278 282 10.1016/0735-6757(87)90350-0 3593492

[B14] Olsson T Terent A Lind L Rapid Emergency Medicine score: a new prognostic tool for in-hospital mortality in nonsurgical emergency department patients J Intern Med 2004 255 5 579 587 10.1111/j.1365-2796.2004.01321.x 15078500

[B15] Widgren BR Jourak M Medical Emergency Triage and Treatment System (METTS): a new protocol in primary triage and secondary priority decision in emergency medicine J Emerg Med 2011 40 6 623 628 10.1016/j.jemermed.2008.04.003 18930373

[B16] Fitzgerald G Jelinik G Scott D Gerdtz MF Emergency department triage revisited Emerg Med J 2010 27 86 92 10.1136/emj.2009.077081 20156855

[B17] Grafstein E Bullard MJ Warren D Unger B CTAS National Working Group Revision of the Canadian Emergency Department Information System (CEDIS) Presenting Complaint List version 1.1 Can J Emerg Med 2008 10 2 151 173 10.1017/s1481803500009878 18371253

[B18] Jorsboe H Schroder M Barylak M Anderson P Inter-observer variation in the triage-process Scand J Trauma Resusc Emerg Med 2010 18 P16 10.1186/1757-7241-18-S1-P16

